# Time-course of heart rate variability after total hip arthroplasty

**DOI:** 10.1007/s10877-023-00992-9

**Published:** 2023-04-13

**Authors:** Mikkel Nicklas Frandsen, Claus Varnum, Nicolai Bang Foss, Jesper Mehlsen, Henrik Kehlet

**Affiliations:** 1 Section for Surgical Pathophysiology, Copenhagen University Hospital, Rigshospitalet, Copenhagen, Denmark; 2 Department of Orthopedic Surgery, Lillebaelt Hospital, Vejle, Denmark; 3 Department of Anesthesia and Intensive Care, Hvidovre University Hospital, Hvidovre, Denmark

**Keywords:** Heart rate variability, Total hip arthroplasty, Enhanced recovery after surgery, Surgical risk stratification, Orthostatic intolerance

## Abstract

Heart rate variability (HRV) is a measure of the autonomic nervous system function and possibly related to postoperative outcome. Despite several HRV studies in different surgical settings, optimal indices and timepoints for measuring have not been adequately determined. Consequently, there is a need for detailed descriptive procedure-specific studies on the time-course of perioperative HRV within a modern fast-track surgical setting. We measured HRV continuously in 24 patients from 4 days before until 9 days after total hip arthroplasty (THA). Statistical methods included mainly ANOVA and t-tests or Kruskal–Wallis and pairwise Wilcoxon test. Patients completed the Orthostatic Discriminant and Severity Scale five times during the study describing autonomic nervous system dysfunction. Standard deviation between normal-to-normal beats and the total power of HRV were reduced for at least 9 days following THA, with a trend towards increased HRV leading up to the day of surgery. The balance between low- and high-frequency power of HRV was reduced in the postoperative evenings. There was increased orthostatic intolerance symptoms on the first postoperative day, with symptoms of pain, fatigue and weakness decreasing after the first postoperative day. Median hospital stay was 1 day. We provide the first detailed description of perioperative time-course of HRV and orthostatic symptoms in fast-track THA, showing reduced HRV after surgery for at least a week, and that HRV changes are sensitive to time of day and timing before and after surgery. These results are helpful in designing future HRV studies in perioperative risk assessment and outcome.

**Supplementary Information:**

The online version contains supplementary material available at 10.1007/s10877-023-00992-9.

## Introduction

Heart rate variability (HRV) is a measure of the autonomic nervous systems control of the heart and is derived from electrocardiographic measures [1]. A multitude of HRV measures exist, and the theoretical and physiological background has been reviewed extensively elsewhere [1, 2].

Decreased HRV may predict cardiac mortality in medical diseases and all-cause mortality following myocardial infarction [3, 4], raising interest in the possible use for risk stratification in surgery.

Preliminary data suggests that HRV may predict short term outcomes in hip fracture patients [5], and persistent pain in knee arthroplasty and carpal tunnel surgeries [6, 7]. Also, a recent systematic review concluded that preoperative HRV can predict intraoperative hypotension and postoperative atrial fibrillation [8]. Despite these interesting early results, it is unclear when HRV should be measured and which indices to choose to optimize the utility of perioperative HRV measurements in surgical risk stratification [8].

Four studies have shown a decrease in HRV after total hip arthroplasty (THA). One study in 16 patients, measured 24 h HRV at 3 points in the early perioperative course. However, the exact timing of measurements before and after surgery varied by several days between patients, and did not include the day before surgery [9], which seems important in predicting postoperative complications [8]. The second study measured HRV continuously in 40 patients from the day before surgery until 3 days after but was not procedure-specific, combining THA and vascular surgery in their results [10]. The third (n = 15) measured from the first preoperative to the fifth postoperative day, but is nearly three decades old and do not report nonlinear analyses [11]. Jans et al. measured 5 min HRV at 3 timepoints from 1 h before surgery until 24 h after surgery, but the study was aimed at understanding HRV in response to postoperative mobilization, not to provide a longitudinal description of HRV after THA [12]. Summarizing, these studies show a decrease in HRV after THA but were either small, not procedure-specific, did not consider that HRV might show circadian variation, only report few HRV indices, or were not designed to elucidate the time-course of HRV after THA.

No studies have monitored continuous HRV earlier than the day before surgery, and during the first week after THA. Furthermore, no continuous HRV studies are available from an established evidence based enhanced recovery setting. As such, the pathophysiology and time course of HRV in a modern fast-track perioperative setting is inadequately described, precluding rational use of HRV for prediction and possible interventions to improve outcome.

Therefore, to better guide and improve the methodology for future studies examining the role of HRV in risk stratification in surgical patients, more information on which timepoints to measure and which indices to use is needed. Accordingly, we aimed to describe continuous HRV measurements starting 4 days before surgery and ending 9 days after in patients undergoing THA in an established fast-track setting, shown to decrease postoperative complications [13].

## Methods

The need for ethical approval was waived by the regional Health research committee Region Syd (case no.: 20/46585, no. 233), and approval for keeping and exchanging data between Danish regions was given by the Danish Data Protection Agency (case no.: 21/10980).

### Patient recruitment and demographics

**Fig. 1:**
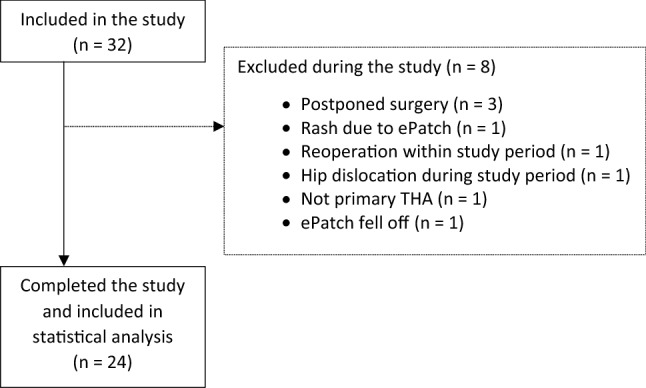
Patient flow chart

Patients were contacted by a research nurse when they were planned for THA at the Department of Orthopedic Surgery at Lillebaelt Hospital—Vejle. Inclusion criteria were: Age ≥ 18 years and scheduled to undergo elective primary THA. The exclusion criteria were: Diabetes mellitus, malignancy, known autonomic dysfunction, cardiac arrhythmias, and pacemaker treatment. 24 patients finished the study as per protocol (Fig. 1) and were included in the statistical analysis. Patient demographics and intraoperative data were gathered from electronic health records or anesthesia charts.

### Perioperative regimen

Patients received either spinal or general anesthesia according to the institution guidelines. All procedures were carried out in a fast-track setting by surgeons with experience in THA surgery. The fast-track protocol includes preoperative tranexamic acid and 125 mg methylprednisolone, standardized fluid management and a mobilization protocol (< 6 h postoperatively). All THAs were carried out through a standard posterolateral approach, and neither drain nor local infiltration analgesia was used. Patients were discharged when the standard functional discharge requirements were met [14].

Oral analgesic regimen started on the morning of surgery with 1 g paracetamol and 400 mg ibuprofen and continued after surgery with 1 g paracetamol combined with 400 mg ibuprofen 4 times daily. 10–20 mg of morphine or other opioids in equivalent doses were used as rescue opioids.

### HRV measurement

4 days before surgery (PRE4), patients had an ePatch 2.0 (Biotelemetry Inc., Sweden) [15] applied onto the skin above the sternum, 1 cm below the jugular notch. The device was set to record a one-channel ECG for 14 days at 256 Hz. The patients wore the ePatch continuously for 14 days, until the ninth postoperative day (POD9), where it was removed and returned to the hospital. There were no physical restrictions when included in the study, but patients were discouraged from submerging the ePatch in water.

### Questionnaire

To describe the impact of surgery on the symptoms of autonomic nervous system dysfunction, patients filled out the orthostatic discriminant and severity scale (ODSS) 5 times during the study: (1) PRE4, (2) day of surgery (DOS) at 6–7 PM, (3) POD1, (4) POD5 and (5) POD9, each time evaluating their symptoms since the last time they filled out the questionnaire. The first questionnaire asked patients to evaluate symptoms in the past year of their life.

We translated the ODSS from English into Danish. The ODSS is a validated 33 point questionnaire with an orthostatic part consisting of 22 questions evaluating orthostatic symptoms, and a non-orthostatic part, with 11 questions on more generalized symptoms (pain, weakness, fatigue) [16]. To describe patient-reported prevalence of orthostatic intolerance, we extracted questions 2 and 25, addressing dizziness upon standing, and when getting out of bed in the morning, respectively.

### Statistical analysis

Heart rate variability analysis was performed using the Cardiscope ANALYTICS—Professional Edition (version 1.3.230) and Kubios HRV Premium (version 3.5.0) software. The recordings were divided into 3 daily periods: NIGHT (11PM to 7AM), DAY (7AM to 3PM) and EVENING (3PM to 11PM). The day started with the NIGHT episode, meaning that for example the NIGHT period of postoperative day 1 (POD1) started at 11PM on the day of surgery. The day period started at 7AM and the EVENING episode started at 3PM on POD1. All statistical analyses were performed in R (version 4.1.1) with R-studio (2022.02.3, Build 492) as the user interface. We were interested in long term HRV only, so for each patients’ recording, periods with less than 10,000 normal-to-normal beats were excluded. In the total data, periods with less than 15 total valid measures, defined as ≥ 15 patients having valid measures in that period, were excluded from analysis. Finally, we excluded the DAY period of the DOS from analysis as we did not control for what specific time of the DAY period patients had surgery, and did not wish to include pre-, intra-, and postoperative measures in the same period data.

The data were checked for normality by visual inspection of Q-Q plots, and extreme outliers identified with the “rstatix package” were removed. During repeated measures analysis, continuous data were automatically tested for sphericity by Mauchly’s test and subsequently underwent automatic Greenhouse–Geisser sphericity correction if they violated the sphericity assumption. ANOVA was performed if the assumptions were correct, followed post-hoc pairwise t-tests. If there were complete data for a given parameter, the t-tests were paired. If the assumptions were violated, we used Friedman test, and post-hoc Wilcoxon signed rank test. For paired categorical data we used Cochran Q test and subsequent pairwise McNemar test. We did not impute missing values. As this was a hypothesis generating study, we decided to perform post-hoc tests regardless of the ANOVA results, but we only report adjusted p-values, which was done by the “Hochberg” method. Significance level was set at p < 0.05. We did not perform a formal power calculation as this was a hypothesis generating exploratory study but two prior studies studying HRV in THA included 15–23 patients in their analyses [11, 12], therefore, we settled on 24.

## Results

### Demographics and perioperative characteristics

Four of 24 patients were discharged on the DOS, and only one patient needed to stay in hospital for more than 1 day. None of the patients experienced cardiovascular complications during or after surgery. Most patients received vasopressors during the surgery (17/24). Sedation was administered on patient request in addition to spinal anesthesia in all but 1 patient (Table 1). Despite low power we, as an exploratory analysis, stratified patients by type of anesthesia. These results are presented in the supplementary material.

**Table 1:** Perioperative characteristics: one patient receiving spinal anesthesia did not receive propofol sedation

Pre-, intra-, and postoperative characteristics	N = 24
Sex	
Female	13 (54%)
Male	11 (46%)
Age/years	65 (9)
BMI/kg/m^2^	27.6 (5.2)
ASA	
1	8 (33%)
2	13 (54%)
3	3 (12%)
Systolic baseline/mmHg	145 (15)
Diastolic baseline/mmHg	86 (9)
Anesthesia	
General	16 (67%)
Spinal	8 (33%)
Intraoperative vasopressors	17 (71%)
Procedure length/min	52 (11)
Combined fluids/ml	2038 (469)
Bleeding/ml	223 (80)
LOS/days	
0	4 (17%)
1	19 (79%)
2	1 (4%)

Data presented as Mean (SD); n (%)

### Heart rate variability

We chose to report data on standard deviation of normal-to-normal intervals (SDNN), total power (TP), low frequency divided by high frequency power (LF/HF) and detrended fluctuation analysis alpha-1 (DFA1) in accordance with a previous publication from our group [8]. Other HRV indices are reported in the supplementary material.

#### Standard deviation of normal-to-normal intervals (SDNN)

**Fig. 2:**
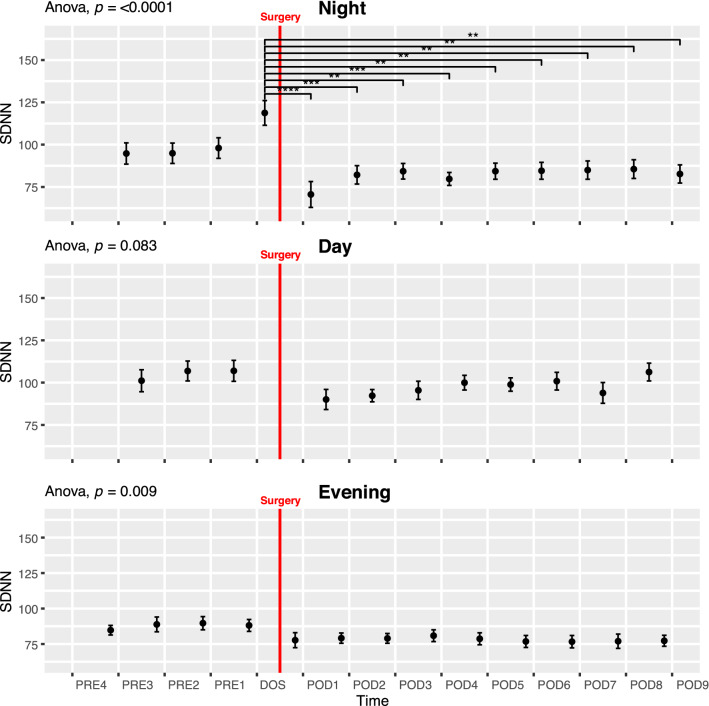
Course of Standard deviation of normal-to-normal beats (SDNN) in the perioperative. One-way ANOVA followed by post-hoc pairwise t-test between timepoints adjusted for mass significance. Subject 6 was removed from SDNN analysis due to being an extreme outlier. **p < 0.01, ***p < 0.001, ****p < 0. 0001. Data presented as mean (dot) ± SE (error bars)

SDNN showed a peak on the night before surgery, and a persistent decrease in the postoperative period in the NIGHT periods (ANOVA p < 0.01; Fig. 2). Pairwise t-test showed SDNN was significantly reduced on all postoperative nights compared to the DOS night (p < 0.01 Fig. 2 Night). This decrease happened in both the general and spinal anesthesia group, but only reached significance in the general anesthesia group (Supplementary Fig. 15 Night). The peak in SDNN was absent during the EVENING periods, but the overall pattern of reduced SDNN following surgery was significant (ANOVA p < 0.01). In the DAY periods, we only found a trend towards decrease after surgery and a subsequent increase towards baseline (ANOVA p = 0.083; Fig. 2 Day). Preoperatively, SDNN showed significant circadian variation (ANOVA p < 0.001; Fig. 6A), most prominent between the evening and night before surgery (p < 0.01; Fig. 6A). When splitting the data by mode of anesthesia the group receiving general anesthesia had significantly higher SDNN on the day before surgery (p < 0.05 Supplementary Fig. 15 Day) and trended towards being higher in ANOVA (p = 0.075, Supplementary Fig. 15 Day) possibly reflecting higher overall variability, but only in the daytime.

#### Total power (TP)

**Fig. 3:**
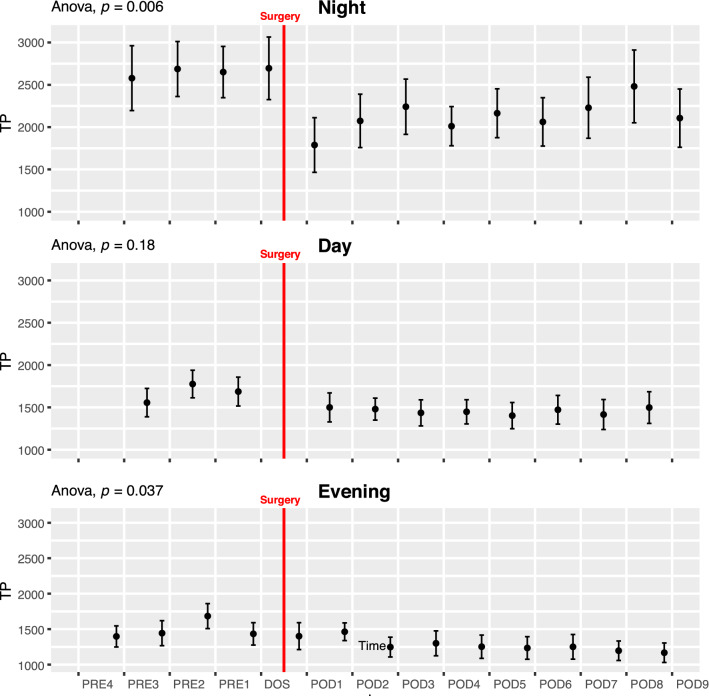
Course Total Power (TP) in the perioperative period. One-way ANOVA showing significant fluctuations in the night and evening periods. Subject 6 was removed from TP analysis due to being an extreme outlier. Data presented as mean (dot) ± SE (error bars)

During EVENING and NIGHT, there was an overall pattern of reduced TP following surgery (ANOVA p < 0.05 Fig. 3 Night and Evening), but insignificant in pairwise t-tests. Preoperatively, TP was significantly higher in the NIGHT periods compared to DAY and EVENING (p < 0.05; Fig. 6B).

#### Low frequency divided by high frequency power (LF/HF)

**Fig. 4:**
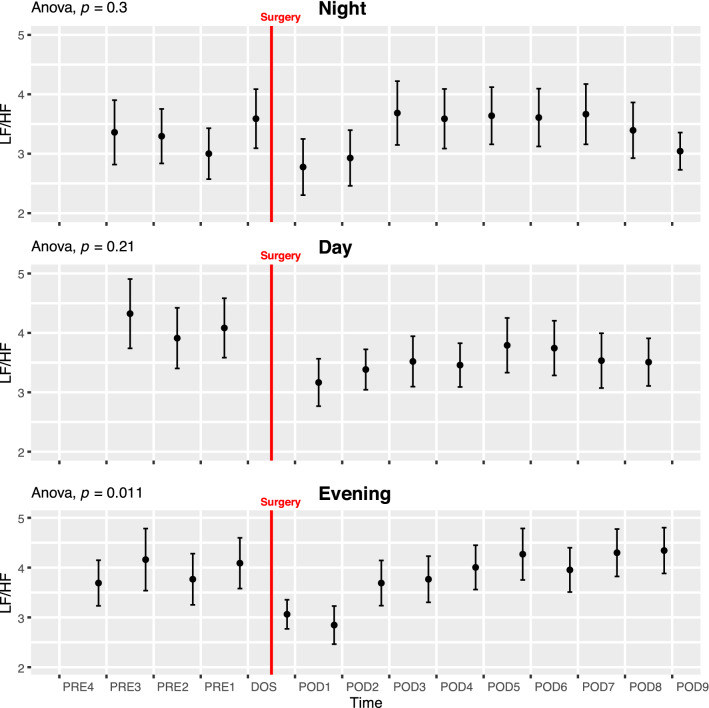
Course of low frequency power divided by high frequency power (LF/HF) in the perioperative period. One-way ANOVA showing significant fluctuations in evenng period. Data presented as mean (dot) ± SE (error bars)

During the EVENING period there was a decrease in LF/HF at DOS and POD1 followed by a return to baseline in the remaining postoperative period (ANOVA p = 0.011; Fig. 4 Evening). Pairwise t-test did not show any significant differences between single timepoints, but we did see a significant circadian variation (p = 0.037; Fig. 6C). After stratifying by anesthesia, LF/HF was always higher in the spinal anesthesia group during the night and day, but it did not reach significance in ANOVA and was only significant on the second preoperative night in t-tests (p < 0.05, Supplementary Fig. 17).

#### Detrended fluctuation analysis α1 (DFA1)

**Fig. 5:**
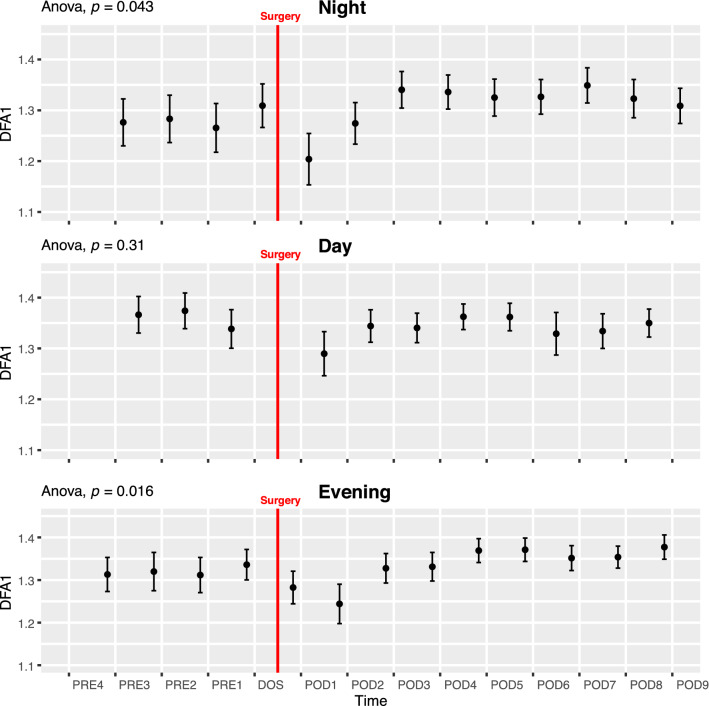
Course of detrended fluctuation analysis alpha-1 (DFA1) in the perioperative period. One-way ANOVA showing significant fluctuations in NIGHT and EVENING periods. Data presented as mean (dot) ± SE (error bars)

**Fig. 6:**
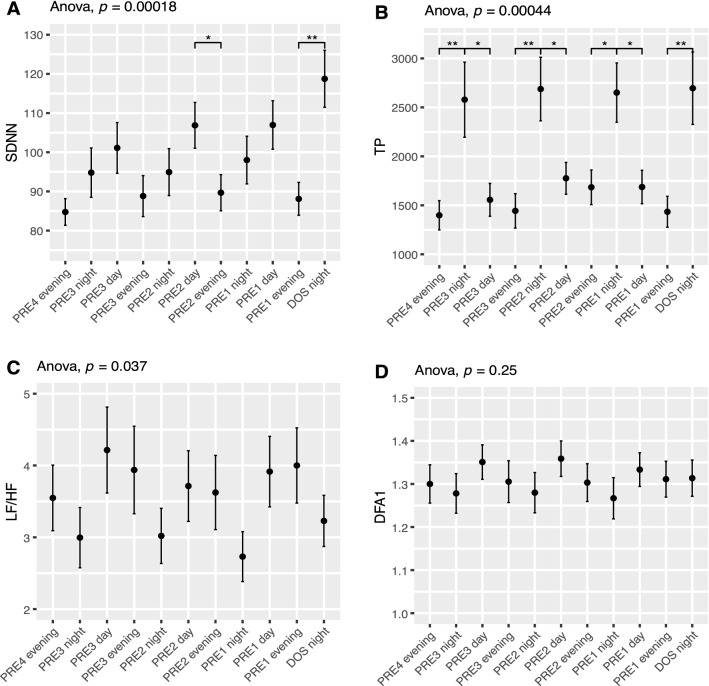
Preoperative time course of **A** SDNN showing significant circadian variation with increases from EVENING to NIGHT to DAY periods, and then a drop to EVENING again, **B** TP showing significantly higher values in the EVENING periods, **C** LF/HF and **D** DFA1 with no significant development during the preoperative period. Subject 6 was removed from SDNN and TP analysis due to being an extreme outlier. Data presented as mean (dot) ± SE (error bars). *p < 0.05, **p < 0.01

DFA1 was significantly reduced during the NIGHT and EVENING periods at POD1 and subsequently returned to baseline (ANOVA p < 0.05; Fig. 5). Post-hoc pairwise t-test did not reveal significant differences between any of the timepoints and there was no significant circadian variation in DFA1 (Fig. 6D).

### Orthostatic discrimination and severity questionnaire

More patients experienced orthostatic intolerance the day after surgery compared to preoperatively (Cochran Q test p < 0.03; Supplementary Fig. 1A, B), defined as either dizziness upon standing or when getting out of bed first in the morning. Post-hoc pairwise McNemar tests revealed a significant increase from DOS to POD1 and a significant decrease from POD1 to POD9, but these results did not retain significance after p-value adjustment (Supplementary Fig. 1A, B).

The patients did not report orthostatic symptoms at baseline, mostly scoring the minimum value. Non-parametric analysis revealed a significant increase in orthostatic symptom burden (Friedman test p < 0.01) with a peak at POD1 during the study period. Subsequent pairwise tests showed significant increase in scores from PRE4 and DOS to POD1 and a decrease from POD1 to POD9 (p < 0.05; Supplementary Fig. 2A).

As an exploratory analysis we analyzed occurrence of self-reported OI, and change in orthostatic symptom score at DOS after surgery or POD1, in relation to HRV based on a previous publication on orthostatic intolerance in a non-surgical setting [17], but found no significant associations.

The non-orthostatic symptom score peaked in the preoperative days and decreased in the days after surgery. (ANOVA p < 0.001; Supplementary Fig. 2B). Subsequent pairwise, paired t-tests showed significant decreases at several points in the study, with the lowest values at POD9, showing that patients experienced significantly less pain, weakness and/or fatigue from 4 to 9 days after surgery.

## Discussion

Our study is the first to report continuous 24 h monitoring of HRV in the days leading up to and for 9 days following total hip arthroplasty in a fast-track setting. We have confirmed that heart rate variability shows dynamic changes from before surgery to several days after. We have shown that these changes are found both in the days leading up to and following surgery, and that they display circadian variations. We found the changes were primarily reflected by a drop in measures of total variability both in time- and frequency domain after surgery and saw trends in indices reflecting decreased baroreceptor control concurrent with postoperative orthostatic intolerance.

In accordance with Marsch et al. [11], we have shown a decrease in SDNN after surgery but in addition we demonstrate that the changes depend on the timing of measurements as this decrease primarily takes place during the DAY and NIGHT periods. We also found that the decrease during the NIGHT periods persists until at least 9 days after surgery. In our study, the change in TP (Fig. 3) was not as pronounced as in the study by Marsch et al. [11] and did not reach significance in pairwise testing, most likely due to the fast-track setup which reduces the consequence of the surgical insult. In contrast to Marsch et al. [11] we found some borderline significant differences between patients undergoing spinal and general anesthesia, most pronounced as higher SDNN and very low frequency in the general anesthesia group during the preoperative days (Supplementary Fig. 15 and 24). This difference could be overlooked if not splitting the data by time of day as well. Most of our reported indices, however, show no significant differences between the two groups. It is possible that only some indices are sensitive to type of anesthesia, but due to possible baseline differences on our groups we are hesitant to conclude further on this. Larger studies are needed to elucidate the differences in perioperative HRV related to type of anesthesia, possibly in a randomized setting to eliminate the preoperative baseline differences we observe between groups in some indices. In the study by Bäcklund et al. [10], only a fourth of the patients underwent THA, the rest had vascular surgery and the authors did not analyze HRV according to the time of day, making direct comparisons difficult. However, they recorded continuously until the third postoperative day and reported decreases in LF/HF, HF, LF and very low frequency power during surgery and on the third postoperative day compared to preoperative values. Using the same indices, we did not see quite as pronounced an effect. In very low frequency power, we saw a significant drop from the DOS in the NIGHT lasting until POD9 in pairwise test, as well as a drop in the postoperative period in ANOVA. We found a significant decrease during the NIGHT in LF but no significant changes in HF during the perioperative course (Supplementary Fig. 6–8).

In a more recent study in THA, Grote et al. [9] measured 24 h HRV from 2 to 8 days before, and 2 to 4 days after surgery. Then they followed HRV until 1 year after surgery at 7 different timepoints starting 27 ± 11 days after surgery. Data were pooled in 4 categories (pre- and perioperative, rehabilitation and long-term recovery) without further detailing the time course of HRV and without reporting when the indices returned to preoperative values. They found decreases in SDNN and TP from pre- to the first postoperative days, with return of all measures to preoperative values from 6 to 52 weeks after surgery. Information on circadian variations were only available on a measure of vagal activity, showing increases in the nighttime, similar to what we see in high frequency variation (Supplementary Fig. 13D).

The discrepancy between ours and the previous studies, could result from differences in methodology and in the surgical fast-track set-up. Splitting our 24 h data in 3 segments revealed circadian differences that are not accounted for in other studies and the fast-track set-up is known to significantly improve recovery and reduce hospital length of stay [13] which could likely impact HRV.

Orthostatic intolerance after surgery (i.e. dizziness when standing up) is a common problem after THA, potentially hindering early discharge [18]. Orthostatic intolerance may be caused by inadequate cerebral blood flow due to an inability to increase heart rate, cardiac contractility, and systemic vascular resistance, all processes governed by the autonomic nervous system. It is possible that preoperative autonomic nervous system monitoring by HRV analysis could identify a subset of patients at increased risk of orthostatic intolerance, thus providing a tool for improving trial design by screening patients and only including those at higher risk of orthostatic intolerance. One trial analyzed 5 min HRV immediately before surgery but found no significant differences in TP and LF/HF in the group experiencing early orthostatic intolerance [12], but they did not evaluate the predictive power, and did not report other HRV measures. Additionally, measuring on the day of surgery might not be optimal, as we have found that several HRV indices, including LF/HF and TP, exhibit perioperative fluctuations (Fig. 6A, D).

Post-induction hypotension is a common problem in surgery and is associated with worse postoperative outcome [19], even in a modern fast track surgery setup. We have previously argued that HRV measurement might predict a subset of patients at risk of intraoperative hypotension, and provide information about patients’ autonomic status necessary for understanding the pathophysiology behind intraoperative hypotension and prevention hereof [20]. Similar considerations may apply to preoperative HRV analysis regarding postoperative orthostatic intolerance, but our study did not have the statistical power for this analysis.

The strengths of this study are: (1) The detailed description of HRV for 14 days, continuously covering more of the pre- and postoperative period than previous studies, (2) To present diurnal variation in perioperative HRV changes, (3) A study in a modern fast-track setting.

Limitations include: (1) Sample size of 24 patients without formal power calculation, (2) Combining patients receiving different types of anesthesia.

In conclusion, we have shown that HRV measures vary significantly during the day and over time in relation to fast-track THA, stressing the importance of prolonged observations to describe the dynamics of the autonomic nervous system in the surgical setting. These observations may be valuable in predicting intraoperative hemodynamic instability, and postsurgical complications.

### Supplementary Information

Below is the link to the electronic supplementary material.

Supplementary file1 (DOCX 13999 kb)

## Data Availability

Anonymized datasets and scripts used for analysis can be obtained by request to the corresponding author.

## References

[1] Electrophysiology Task Force of the European Society of Cardiology the North American Society of Pacing (1996). Heart rate variability: standards of measurement, physiological interpretation, and clinical use. Circulation.

[2] Henriques T, Ribeiro M, Teixeira A, Castro L, Antunes L, Costa-Santos C (2020). Nonlinear methods most applied to heart-rate time series: a review. Entropy.

[3] Kleiger RE, Miller JP, Bigger JT, Moss AJ (1987). Decreased heart rate variability and its association with increased mortality after acute myocardial infarction. Am J Cardiol.

[4] Mäkikallio TH, Barthel P, Schneider R, Bauer A, Tapanainen JM, Tulppo MP (2005). Prediction of sudden cardiac death after acute myocardial infarction: role of Holter monitoring in the modern treatment era. Eur Heart J.

[5] Ernst G, Watne LO, Frihagen F, Wyller TB, Dominik A, Rostrup M (2017). Decreases in heart rate variability are associated with postoperative complications in hip fracture patients. PLoS ONE.

[6] Bossmann T, Brauner T, Wearing S, Horstmann T (2017). Predictors of chronic pain following total knee replacement in females and males: an exploratory study. Pain Manag.

[7] Nielsen R, Nikolajsen L, Krøner K, Mølgaard H, Vase L, Jensen TS (2015). Pre-operative baroreflex sensitivity and efferent cardiac parasympathetic activity are correlated with post-operative pain. Acta Anaesthesiol Scand.

[8] Frandsen MN, Mehlsen J, Foss NB, Kehlet H (2022). Preoperative heart rate variability as a predictor of perioperative outcomes: a systematic review without meta-analysis. J Clin Monit Comput.

[9] Grote V, Levnajić Z, Puff H, Ohland T, Goswami N, Frühwirth M (2019). Dynamics of vagal activity due to surgery and subsequent rehabilitation. Front Neurosci.

[10] Bäcklund M, Toivonen L, Tuominen M, Pere P, Lindgren L (1999). Changes in heart rate variability in elderly patients undergoing major noncardiac surgery under spinal or general anesthesia. Reg Anesth Pain Med.

[11] Marsch SCU, Skarvan K, Schaefer H-G, Naegeli B, Paganoni R, Castelli I (1994). Prolonged decrease in heart rate variability after elective hip arthroplasty. Br J Anaesth.

[12] Jans Ø, Brinth L, Kehlet H, Mehlsen J (2015). Decreased heart rate variability responses during early postoperative mobilization–an observational study. BMC Anesthesiol.

[13] Petersen PB, Kehlet H, Jørgensen CC (2020). Improvement in fast-track hip and knee arthroplasty: a prospective multicentre study of 36,935 procedures from 2010 to 2017. Sci Rep.

[14] Husted H, Lunn TH, Troelsen A, Gaarn-Larsen L, Kristensen BB, Kehlet H (2011). Why still in hospital after fast-track hip and knee arthroplasty?. Acta Orthop.

[15] Saadi DB, Sørensen HBD, Hansen IH, Egstrup K, Jennum P, Hoppe K (2014). ePatch®—a clinical overview.

[16] Baker J, Paturel JR, Sletten DM, Low PA, Kimpinski K (2018). Initial validation of symptom scores derived from the orthostatic discriminant and severity scale. Clin Auton Res.

[17] Swai J, Hu Z, Zhao X, Rugambwa T, Ming G (2019). Heart rate and heart rate variability comparison between postural orthostatic tachycardia syndrome versus healthy participants; a systematic review and meta-analysis. BMC Cardiovasc Disord.

[18] Jans Ø, Kehlet H (2017). Postoperative orthostatic intolerance: a common perioperative problem with few available solutions. Can J Anaesth.

[19] Wickham AJ, Highton DT, Clark S, Fallaha D, Wong DJN, Martin DS (2022). Treatment threshold for intra-operative hypotension in clinical practice: a prospective cohort study in older patients in the UK. Anaesthesia.

[20] Frandsen MN, Mehlsen J, Bang Foss N, Kehlet H (2022). Pre-operative autonomic nervous system function—a missing link for post-induction hypotension?. Anaesthesia.

