# Supplementary material for: Time-course of heart rate variability after total hip arthroplasty
Source: J Clin Monit Comput. 2023 Apr 13;38(2):423–32. doi: 10.1007/s10877-023-00992-9 (PMC10995030; doi:10.1007/s10877-023-00992-9)
Supplement: Supplementary file 1 — Supplementary file1 (DOCX 13999 kb) [file 10877_2023_992_MOESM1_ESM.docx]

Supplementary figures

Time-course of heart rate variability after total hip arthroplasty


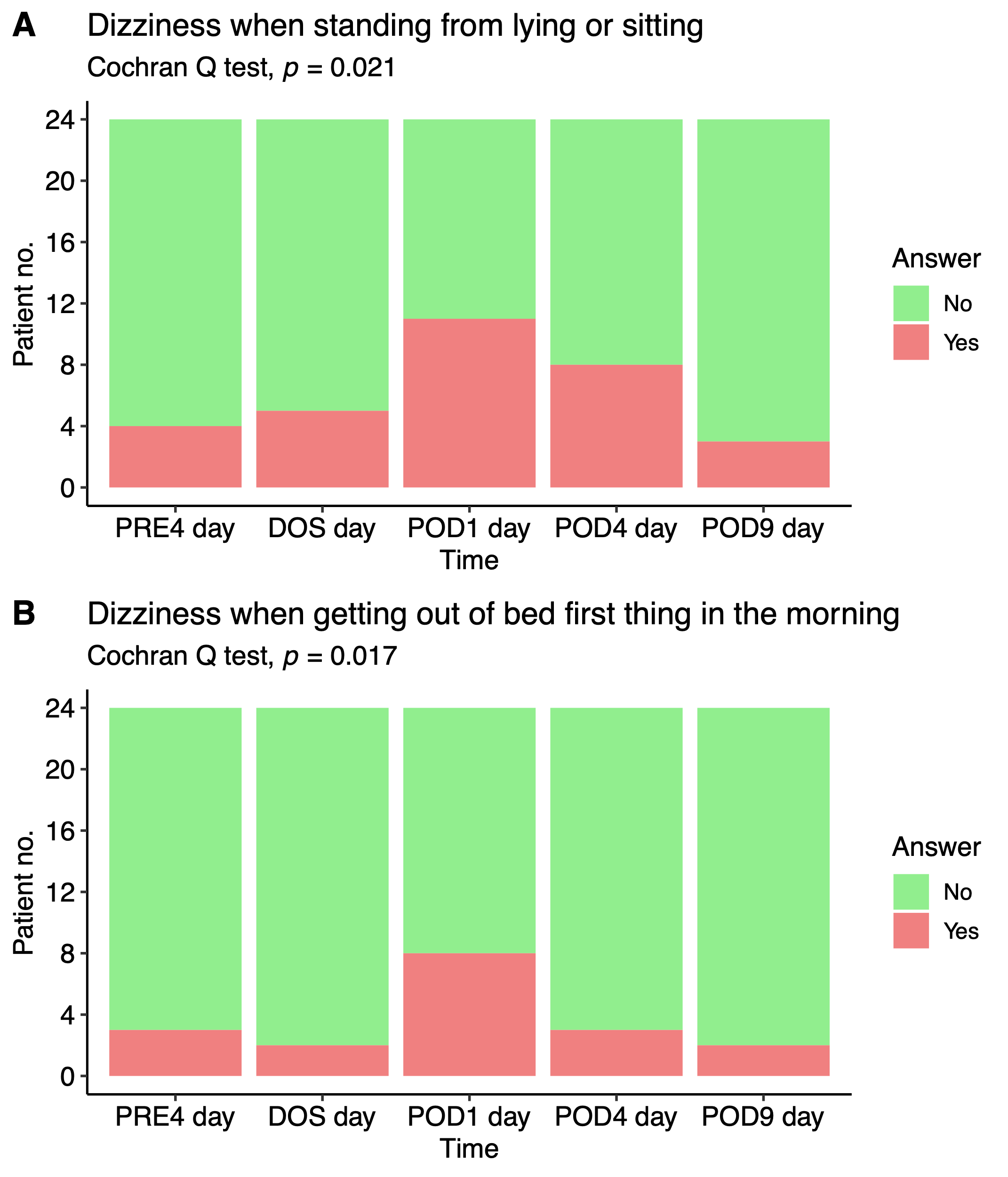


**Supplementary figure 1:** Patients experiencing dizziness when A) standing up, and B) when getting out of bed first thing in the morning. Cochran Q test showing increase to POD1 and then a drop to POD9. Pairwise McNemar test without significant results in both A and B.

**Supplementary figure 2**: A) Orthostatic symptom score (OS) comprised of 22 questions from the ODSS. Friedman test with post-hoc Wilcoxon sign rank test. Increase in orthostatic burden to POD1 and then a drop to POD9, B) Non-orthostatic symptom score (NS) comprised of 11 questions from the ODSS. One-way ANOVA followed by post-hoc paired t-test between timepoints, adjusted for mass significance. *p<0.05, ***p<0.001, ****p<0.0001.


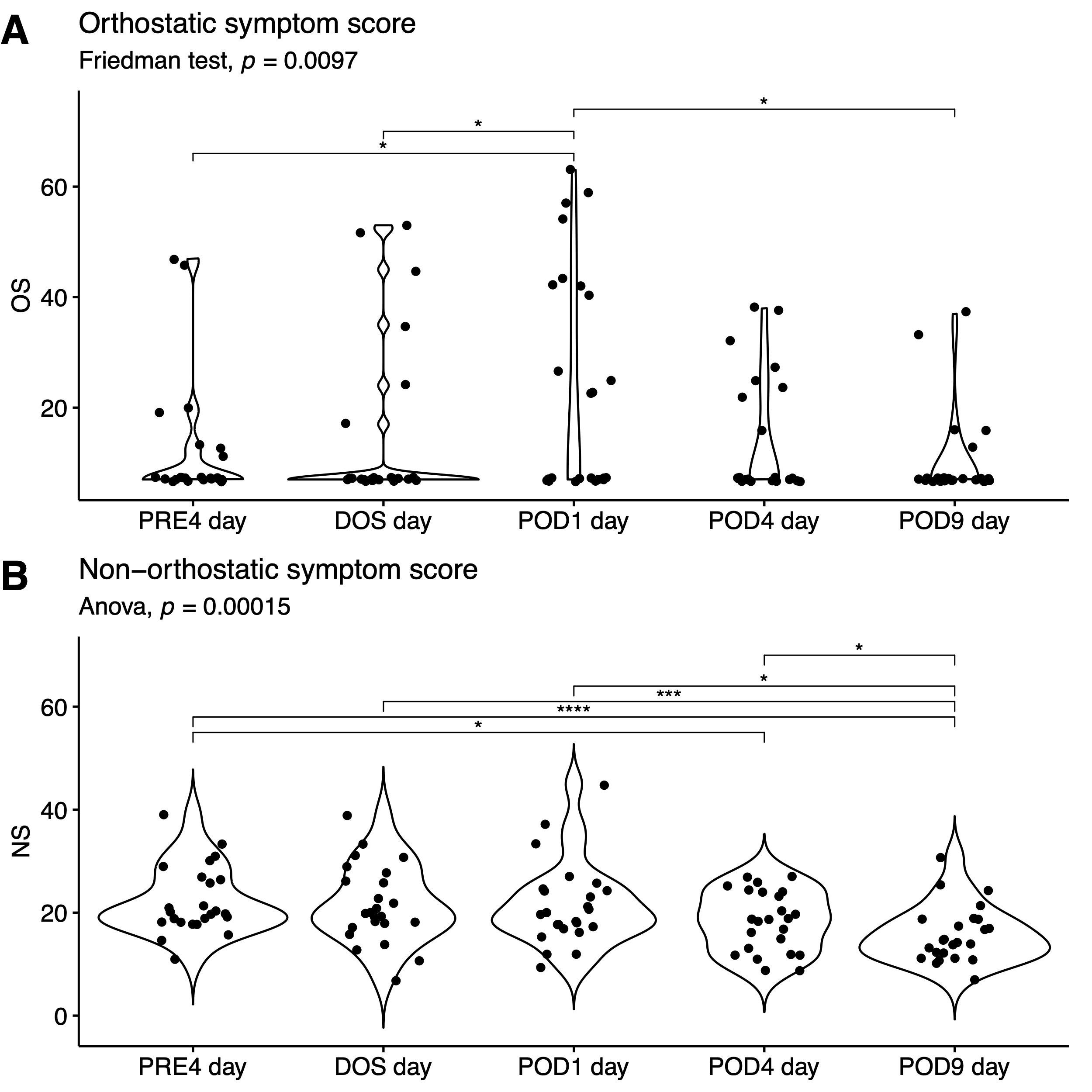

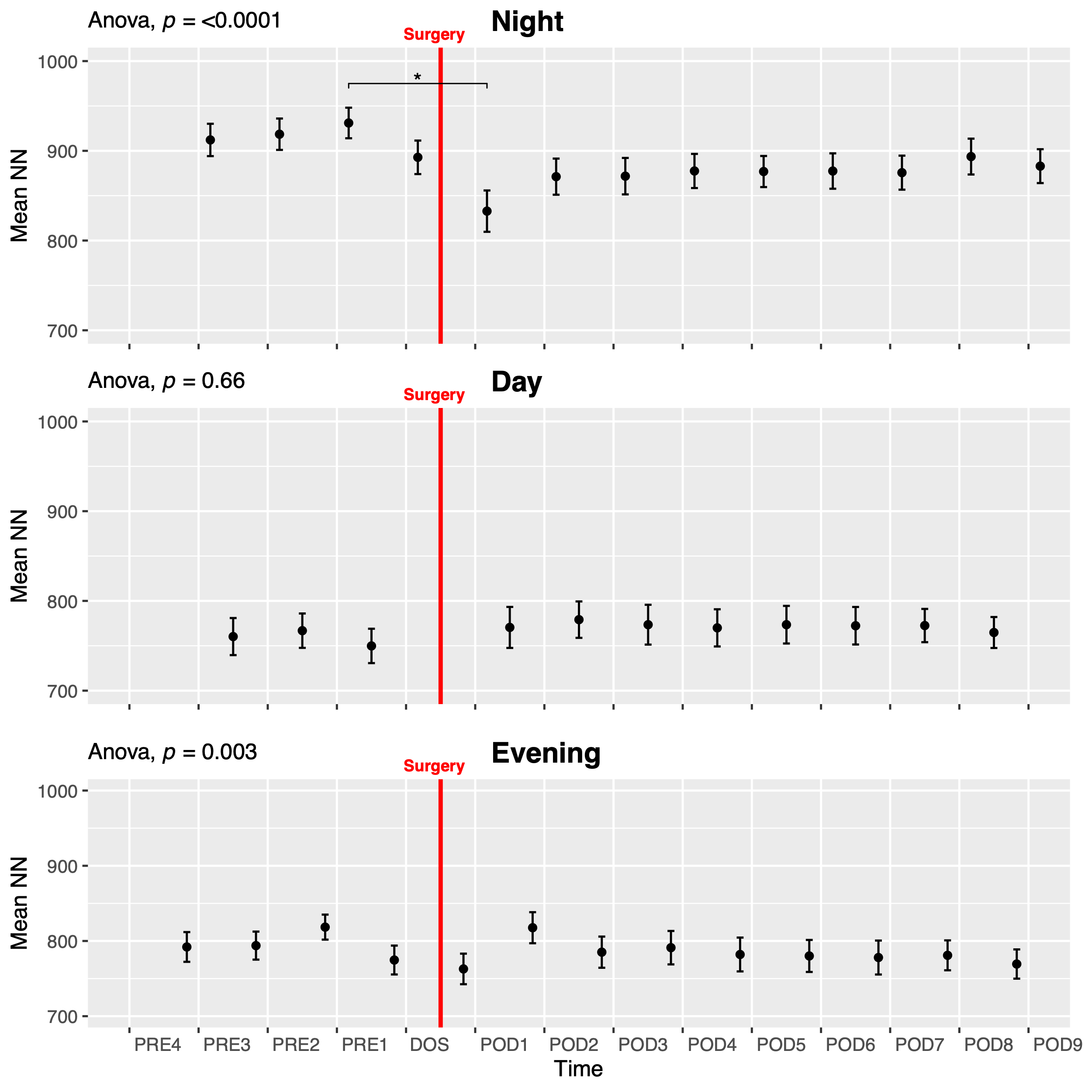


**Supplementary figure 3**: Course of mean NN interval (ms between heartbeats) in the perioperative period. One-way ANOVA followed by post-hoc pairwise t-test between timepoints adjusted for mass significance, showing significantly faster heart rate at first postoperative night compared to PRE1. *p<0.05. Data presented as mean (dot) ± SE (error bars).


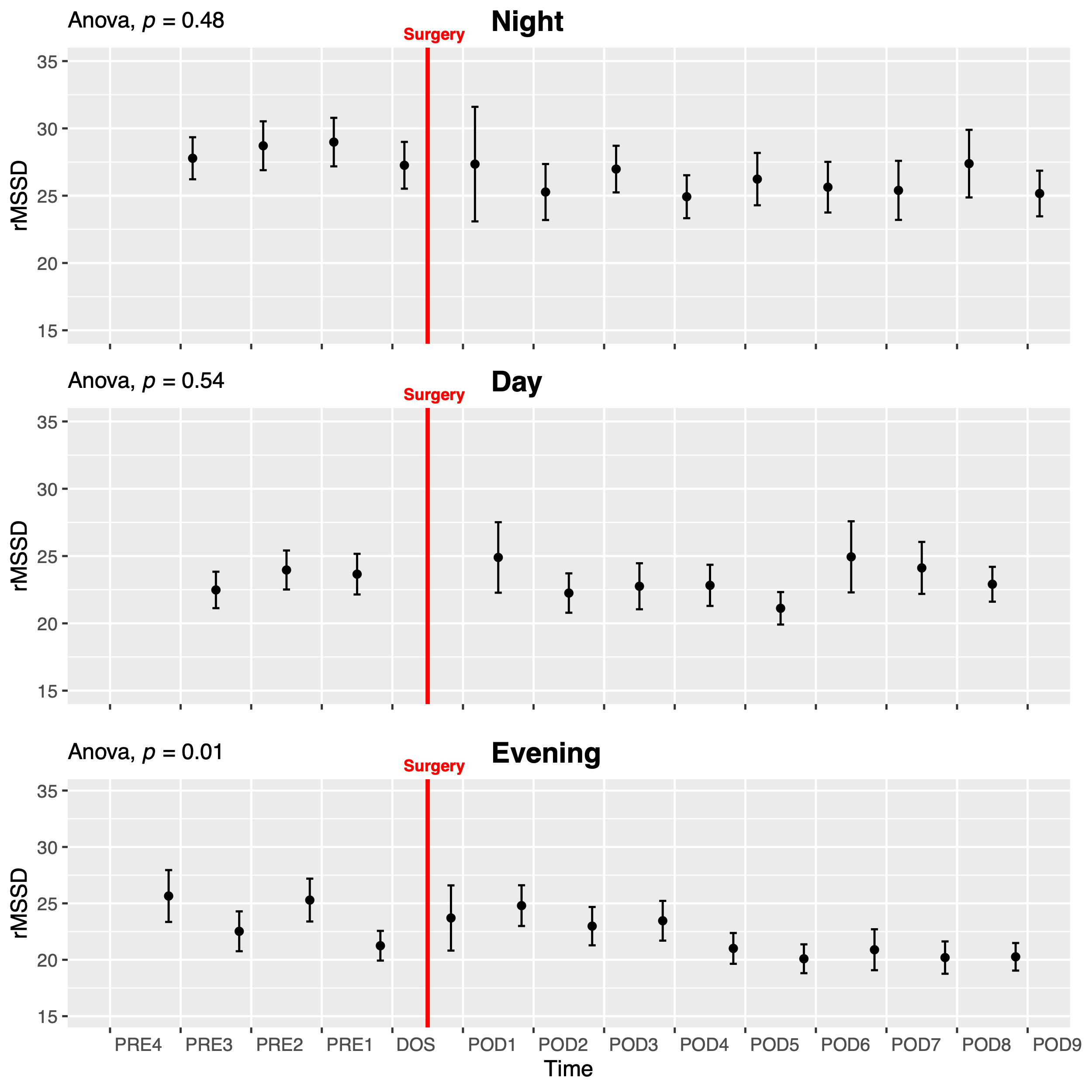


**Supplementary figure 4**: Course of the root mean square of the squared differences between adjacent NN intervals (rMSSD) in the perioperative period. Subjects 6 and 13 were removed from analysis due to being extreme outliers. One-way ANOVA followed by post-hoc pairwise t-test between timepoints adjusted for mass significance. Data presented as mean (dot) ± SE (error bars).


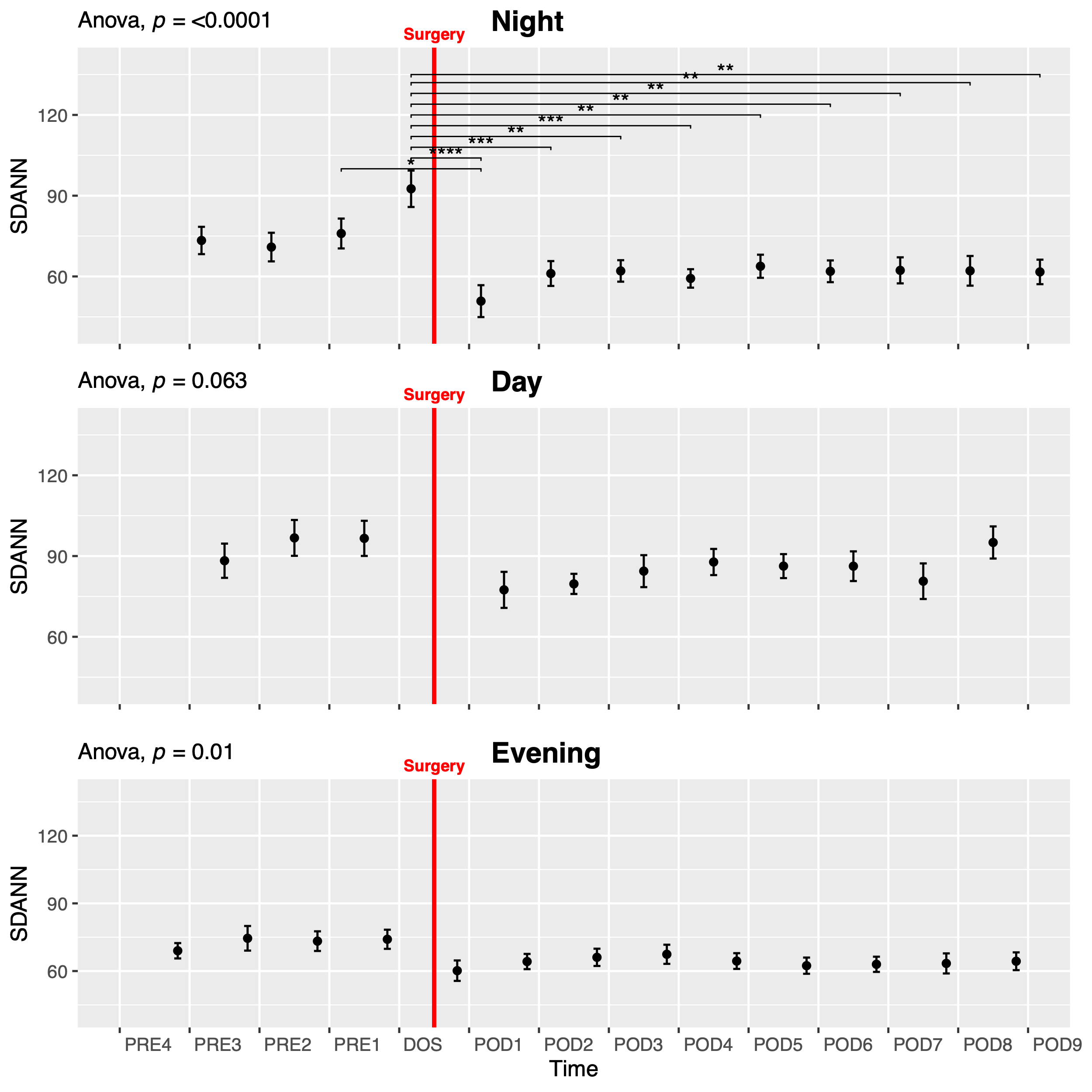


**Supplementary figure 5**: Course of standard deviation of average NN intervals in 5 min segments (SDANN) during the perioperative period. One-way ANOVA followed by post-hoc pairwise t-test between timepoints adjusted for mass significance. Subject 6 was removed from analysis due to being an extreme outlier *p<0.05, **p<0.01, ***p<0.001, ****p<0.0001. Data presented as mean (dot) ± SE (error bars).


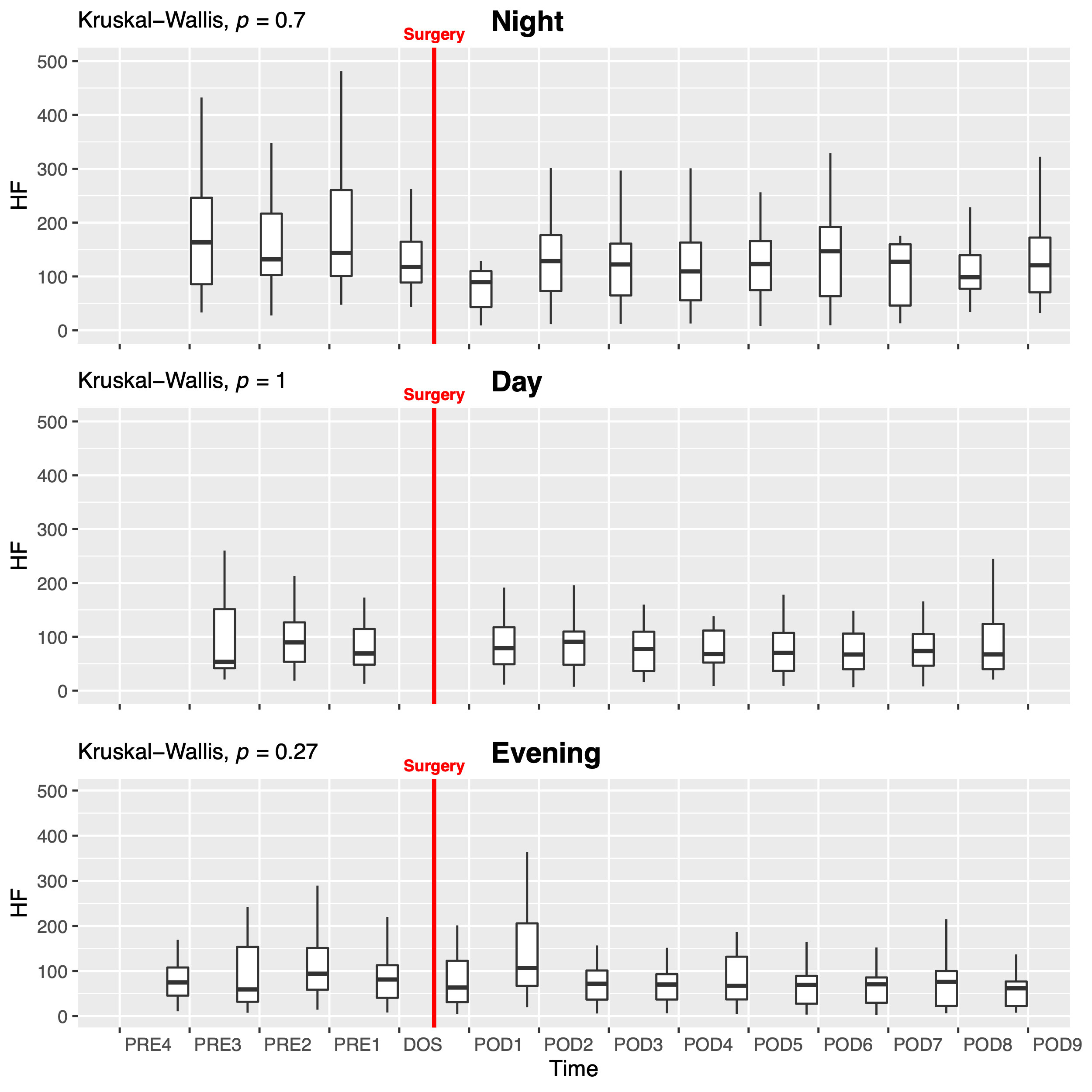


**Supplementary figure 6**: Course of high frequency (HF) in the perioperative period. Due to HF being non-normally distributed, Kruskal-Wallis test was performed, followed by post-hoc pairwise Wilcoxon test between timepoints, adjusted for mass significance and data presented as boxplot. Extreme outliers have been removed from the plot to improve visual quality but is included in analysis.


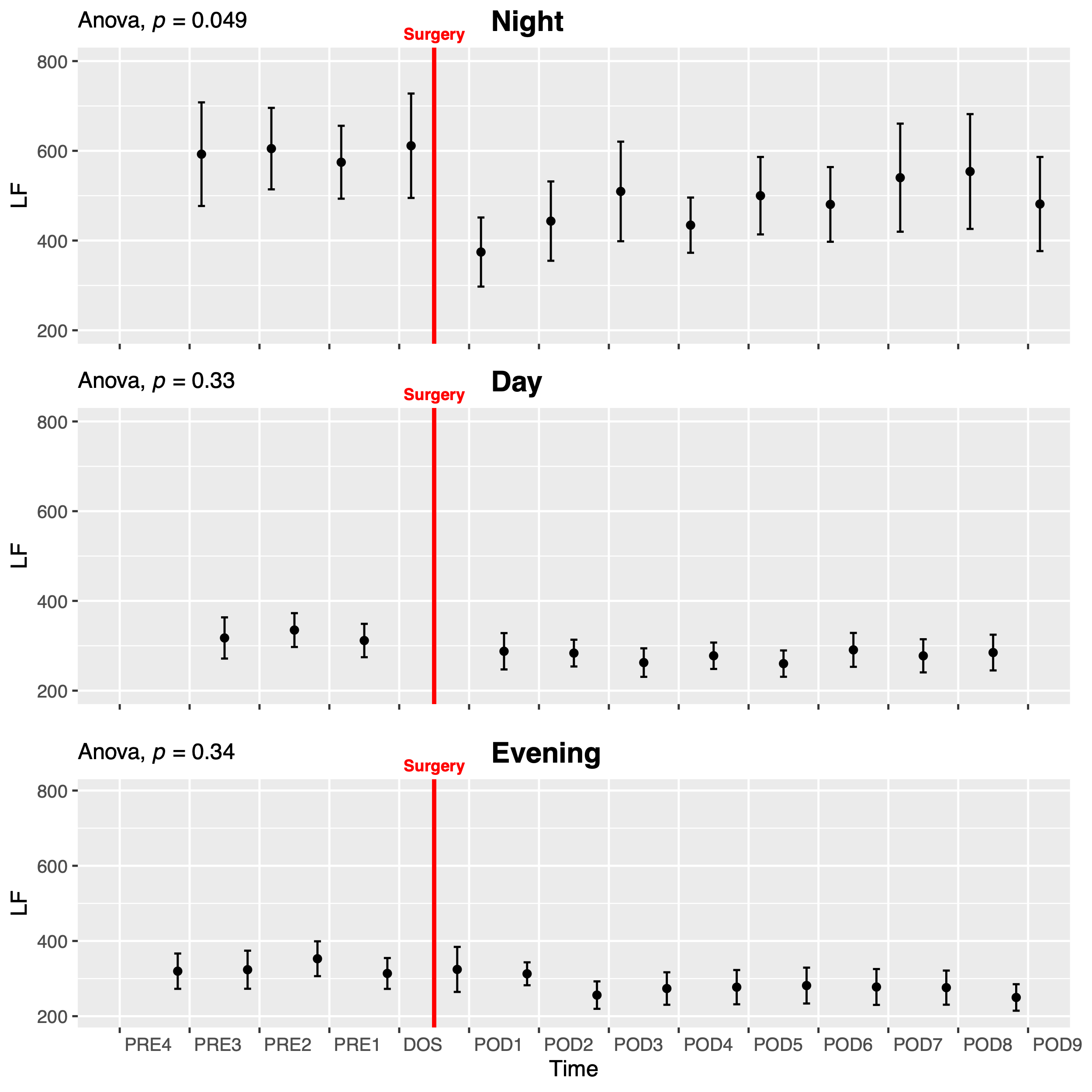


**Supplementary figure 7**: Course of low frequency (LF) power in the perioperative period. One-way ANOVA followed by post-hoc pairwise t-test between timepoints adjusted for mass significance. Subject 6 removed from analysis due to being an extreme outlier. Data presented as mean (dot) ± SE (error bars).


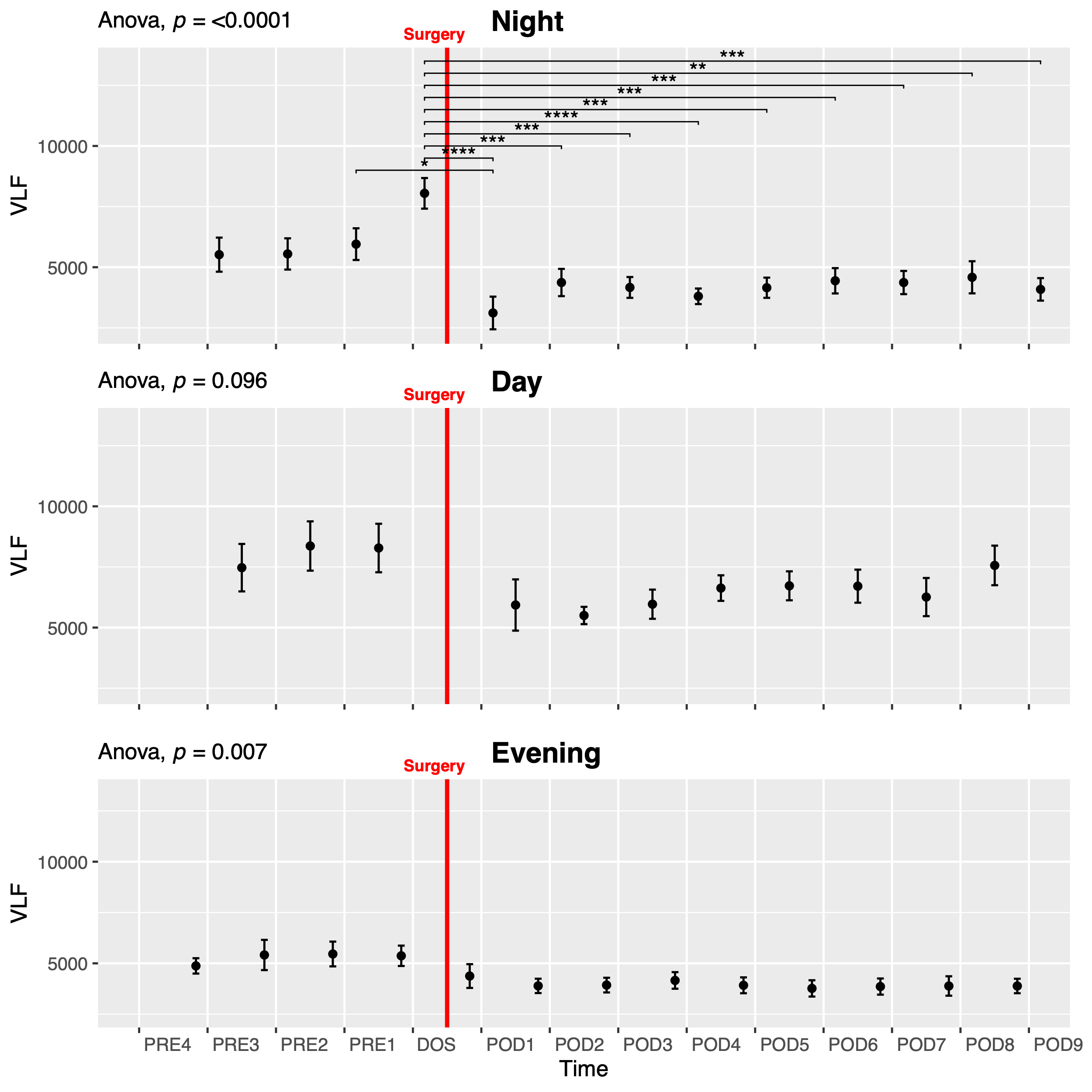


**Supplementary figure 8**: Course of very low frequency power (VLF) in the perioperative period. One-way ANOVA followed by post-hoc pairwise t-test between timepoints adjusted for mass significance. Subjects 6 and 19 removed from analysis due to being extreme outliers. *p<0.05, **p<0.01, ***p<0.001, ****p<0.0001. Data presented as mean (dot) ± SE (error bars).


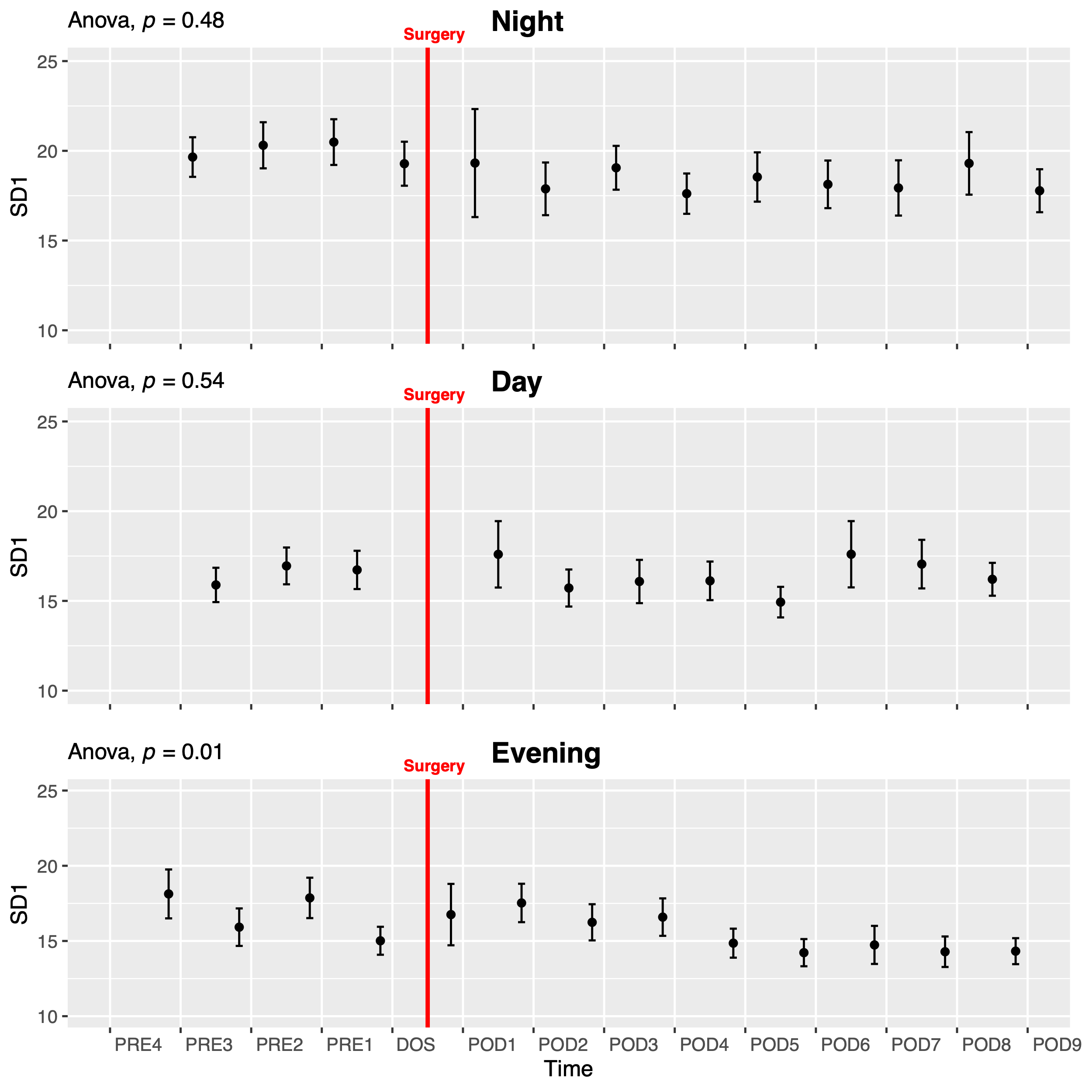


**Supplementary figure 9**: Course of nonlinear SD1 in the perioperative period. One-way ANOVA followed by post-hoc pairwise t-test between timepoints adjusted for mass significance. Data presented as mean (dot) ± SE (error bars).


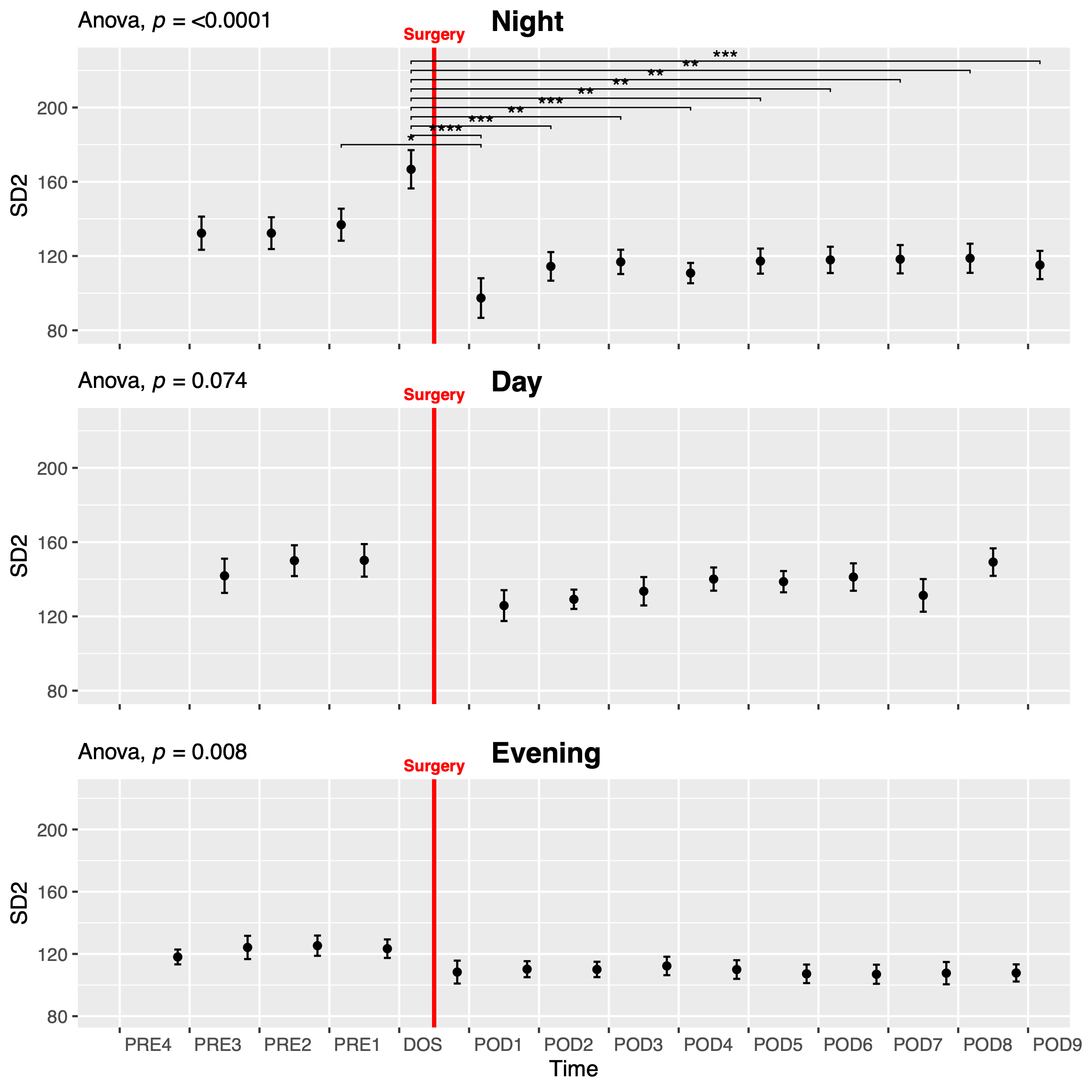


**Supplementary figure 10**: Course of nonlinear SD2 in the perioperative period. One-way ANOVA followed by post-hoc pairwise t-test between timepoints adjusted for mass significance. Subject 6 removed from analysis due to being an extreme outlier. *p<0.05, **p<0.01, ***p<0.001, ****p<0.0001. Data presented as mean (dot) ± SE (error bars).


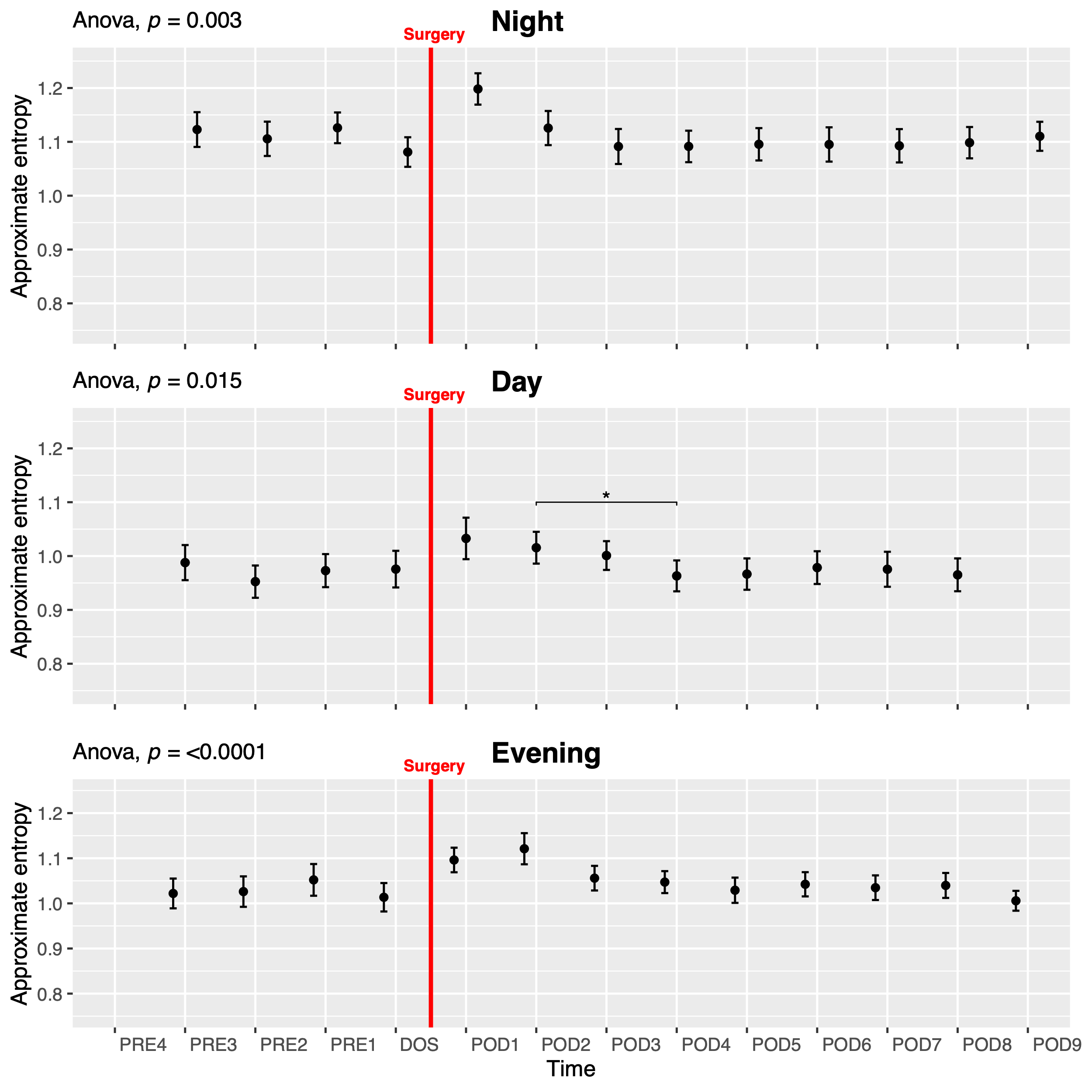


**Supplementary figure 11**: Course of approximate entropy in the perioperative period. One-way ANOVA followed by post-hoc pairwise t-test between timepoints adjusted for mass significance. *p<0.05. Data presented as mean (dot) ± SE (error bars).


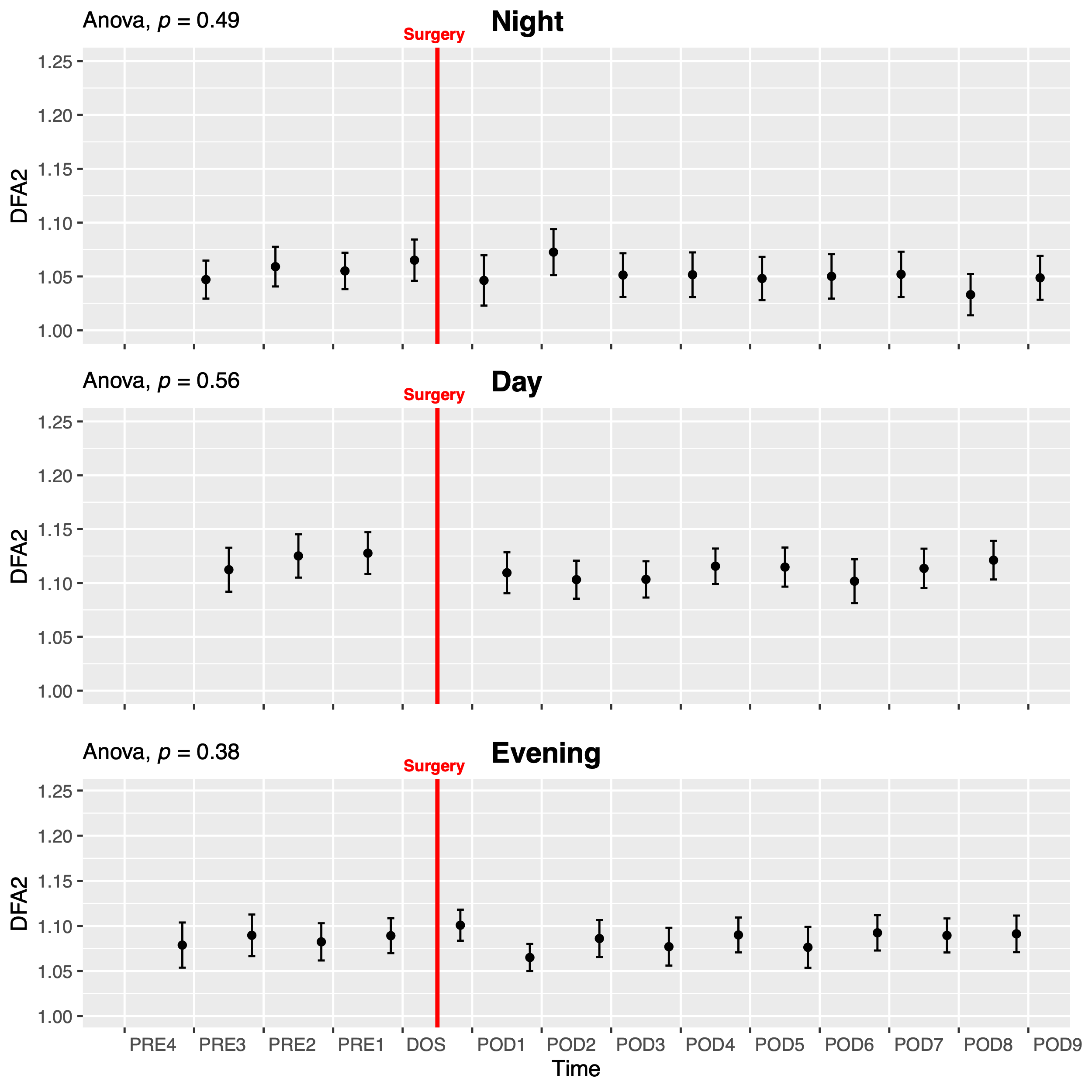


**Supplementary figure 12**: Course of detrended fluctuations analysis α2 (DFA2) in the perioperative period. One-way ANOVA followed by post-hoc pairwise t-test between timepoints adjusted for mass significance. Data presented as mean (dot) ± SE (error bars).


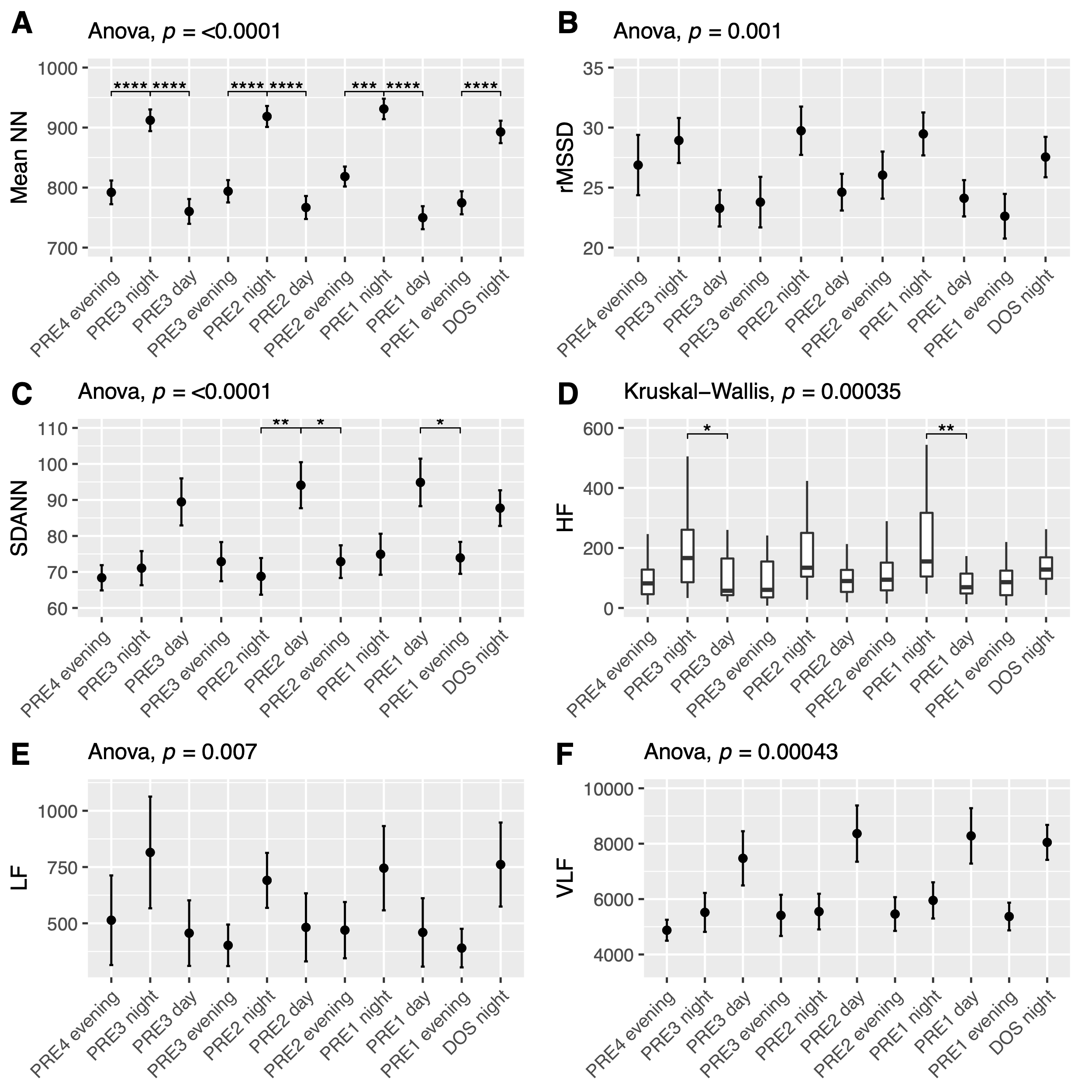


**Supplementary figure 13**: Preoperative time course of A) Mean NN showing significant circadian variation being higher in the NIGHT periods, B) rMSSD showing higher values in the NIGHT periods in ANOVA, C) SDANN showing significantly higher values in the two day-periods leading up to the surgery, D) HF Significantly higher on PRE3 and PRE1 night periods in non-parametric analysis, E) LF showing higher values in the NIGHT periods in ANOVA, F) VLF showing higher values in the NIGHT periods in ANOVA. Subject 6 was removed from rMSSD, SDANN, LF and VLF analysis, Subject 13 was removed from rMSSD and Subject 19 was removed from VLF analysis due to being extreme outliers. Data presented as mean (dot) ± SE (error bars). *p<0.05, **p<0.01, ***p<0.001. **** p<0.0001.


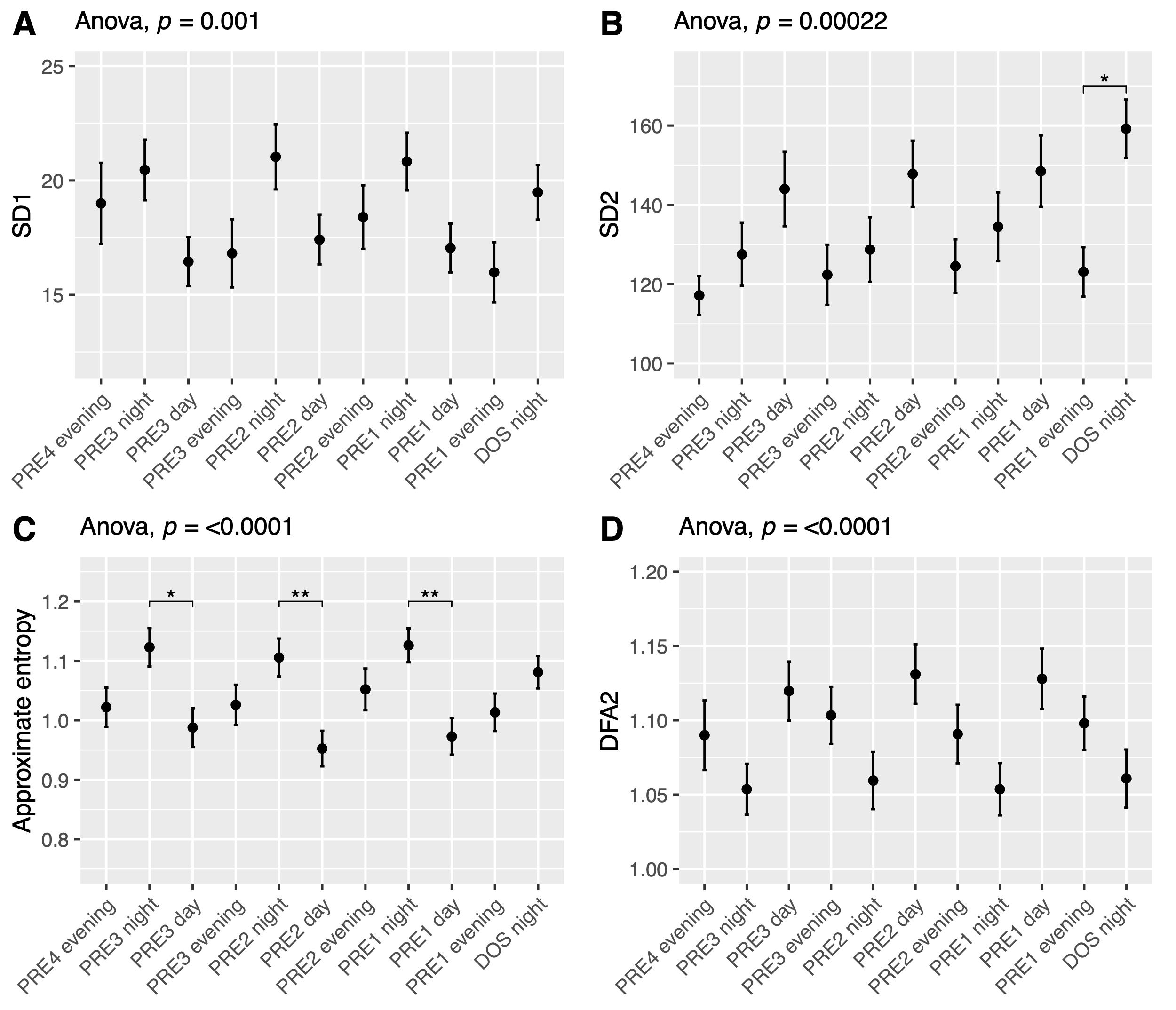


**Supplementary figure 14**: Preoperative time course of A) SD1 showing higher values in the NIGHT periods in ANOVA, B) SD2 showing increase in values towards the DAY periods in ANOVA, C) Approximate entropy showing significantly higher values in the NIGHT compared to DAY periods and D) DFA2 showing decreases towards the NIGHT periods in ANOVA. Subject 6 was removed from SD1, SD2, and subject 13 was removed from SD1 analysis due to being extreme outliers. Data presented as mean (dot) ± SE (error bars). *p<0.05, **p<0.01.

**Supplementary figure 15**: SDNN in the perioperative period, split up by mode of anesthesia Two-way ANOVA followed by post-hoc pairwise t-test between groups. Insignificant ANOVA in all times of day for effect of anesthesia, but intravenous anesthesia group (IV) showed higher SDNN the day before surgery (PRE1) in pairwise t-test. *p<0.05, **p<0.01, ***p<0.001. Data presented as mean (dot) ± SE (error bars). Subject 6 was removed from analysis due to being an outlier.

**Supplementary figure 16**: TP in the perioperative period, split up by mode of anesthesia Two-way ANOVA followed by post-hoc pairwise t-test between groups. Insignificant ANOVA in all times of day for effect of anesthesia, and no significant differences between groups at any single timepoints in pairwise t-test. *p<0.05. Data presented as mean (dot) ± SE (error bars). Subject 6 was removed from analysis due to being an outlier.

**Supplementary figure 17**: LF/HF in the perioperative period, split up by mode of anesthesia Two-way ANOVA followed by post-hoc pairwise t-test between groups. Insignificant ANOVA in all times of day for effect of anesthesia. Significantly higher LF/HF in the night period two days before surgery (PRE2) in pairwise t-test. *p<0.05. Data presented as mean (dot) ± SE (error bars).

**Supplementary figure 18**: DFA1 in the perioperative period, split up by mode of anesthesia Two-way ANOVA followed by post-hoc pairwise t-test between groups. Insignificant ANOVA in all times of day for effect of anesthesia, and no significant differences between groups at any single timepoints in pairwise t-test. Data presented as mean (dot) ± SE (error bars). Subject 13 was removed from analysis due to being an outlier.

**Supplementary figure 19**: Mean NN (time between heart beats in ms) in the perioperative period, split up by mode of anesthesia Two-way ANOVA followed by post-hoc pairwise t-test between groups. Insignificant ANOVA in all times of day for effect of anesthesia. Significant differences between groups on the 6^th^ postoperative night in pairwise t-test. *p<0.05. Data presented as mean (dot) ± SE (error bars).

**Supplementary figure 20**: rMSSD in the perioperative period, split up by mode of anesthesia Two-way ANOVA followed by post-hoc pairwise t-test between groups. Insignificant ANOVA in all times of day for effect of anesthesia, but significantly higher rMSSD on the evening of the day after surgery in pairwise t-test. *p<0.05. Data presented as mean (dot) ± SE (error bars). Subject 6 was removed from analysis due to being an outlier. Subjects 6 and 13 were removed due to being outliers.

**Supplementary figure 21**: SDANN in the perioperative period, split up by mode of anesthesia Two-way ANOVA followed by post-hoc pairwise t-test between groups. Insignificant ANOVA in all times of day for effect of anesthesia, but significantly higher SDANN the day before surgery in pairwise t-test. *p<0.05, **p<0.01, ***p<0.001. Data presented as mean (dot) ± SE (error bars). Subject 6 was removed from analysis due to being an outlier. Subject 6 was removed due to being an outlier.

**Supplementary figure 22**: HF in the perioperative period, split up by mode of anesthesia Kruskal-Wallis test followed by post-hoc pairwise Wilcoxon test between groups. Significant Kruskal-Wallis for effect of mode of anesthesia in the day period, but no significant differences between groups at any single timepoints in pairwise t-test. Data presented as mean (dot) ± SE (error bars).

**Supplementary figure 23**: LF in the perioperative period, split up by mode of anesthesia Two-way ANOVA followed by post-hoc pairwise t-test between groups. Insignificant ANOVA in all times of day for effect of anesthesia, and no significant differences between groups at any single timepoints in pairwise t-test. Data presented as mean (dot) ± SE (error bars). Subject 6 was removed from analysis due to being an outlier.

**Supplementary figure 24**: VLF in the perioperative period, split up by mode of anesthesia Two-way ANOVA followed by post-hoc pairwise t-test between groups. Insignificant ANOVA in all times of day for effect of anesthesia, but significantly lower VLF in the spinal group on the day before surgery in pairwise t-test. *: p<0.05, **p<0.01, ***p<0.001. Data presented as mean (dot) ± SE (error bars). Subjects 6 and 19 were removed from analysis due to being extreme outliers.

**Supplementary figure 25**: rMSSD in the perioperative period, split up by mode of anesthesia Two-way ANOVA followed by post-hoc pairwise t-test between groups. Insignificant ANOVA in all times of day for effect of anesthesia, but significantly higher rMSSD on the evening of the day after surgery in pairwise t-test. *p<0.05. Data presented as mean (dot) ± SE (error bars). Subjects 6 and 13 were removed due to being outliers.

**Supplementary figure 26**: LF in the perioperative period, split up by mode of anesthesia Two-way ANOVA followed by post-hoc pairwise t-test between groups. Insignificant ANOVA in all times of day for effect of anesthesia but significantly higher SD2 in the intravenous anesthesia (IV) group on the day before surgery in pairwise t-test. *: p<0.05, **p<0.01, ***p<0.001.. Data presented as mean (dot) ± SE (error bars). Subject 6 was removed from analysis due to being an outlier.

**Supplementary figure 27**: ApEn in the perioperative period, split up by mode of anesthesia Two-way ANOVA followed by post-hoc pairwise t-test between groups. Insignificant ANOVA in all times of day for effect of anesthesia, and no significant differences between groups at any single timepoints in pairwise t-test. Data presented as mean (dot) ± SE (error bars).

**Supplementary figure 28**: DFA2 in the perioperative period, split up by mode of anesthesia Two-way ANOVA followed by post-hoc pairwise t-test between groups. Insignificant ANOVA in all times of day for effect of anesthesia, and no significant differences between groups at any single timepoints in pairwise t-test. Data presented as mean (dot) ± SE (error bars).
